# Case Report: Defining Applicant Attributes to Be Prioritized in the Selection of Child and Adolescent Psychiatry Subspecialty Residents at the University of Toronto

**DOI:** 10.3389/fpsyt.2021.650317

**Published:** 2021-04-20

**Authors:** Chetana A. Kulkarni, Raj Rasasingham, Nicole N. Woods, Daniel A. Gorman, Peter Szatmari, Mark D. Hanson

**Affiliations:** ^1^Department of Psychiatry, University of Toronto, Toronto, ON, Canada; ^2^Department of Psychiatry, Hospital for Sick Children (SickKids), University of Toronto, Toronto, ON, Canada; ^3^Humber River Regional Hospital, Toronto, ON, Canada; ^4^The Wilson Centre, University Health Network, Toronto, ON, Canada; ^5^Centre for Addiction & Mental Health, Toronto, ON, Canada

**Keywords:** medical education, psychiatry, attributes, admissions, selection, child and adolescent psychiatry

## Abstract

**Background/Objectives:** The child and adolescent psychiatry (CAP) subspecialty training program at the University of Toronto was among the first fully accredited CAP programs in Canada. As one of Canada's largest CAP subspecialty programs, we attract many excellent applicants annually. While objectivity and transparency in the selection of candidates have been valued, it was unclear which applicant attributes should be prioritized. This quality improvement project was undertaken to identify the key applicant attributes that should be prioritized for admission to the program.

**Materials/Methods:** An initial list of attributes was compiled by project team members and feedback solicited. Through iterative design, this list was categorized into “end products,” “branding attributes” and “generic attributes.” The “end products” were removed as these represented outputs of training rather than attributes on which applicant selection should be based. Subsequent steps involved only the “branding” and “generic” attributes. A consensus-building exercise led to the creation of two short-lists of five attributes within each category. Finally, a paired-comparison forced choice methodology was used to determine the ranking of these attributes in order of importance when assessing applicants.

**Results:** The final lists of “generic” and “branding” attributes developed through a consensus-building exercise are presented in rank order based on the paired-comparison methodology. The overall response rate for the forced choice electronic survey was 49% of faculty and learners.

**Conclusions/Discussion:** This project used an iterative process of consensus building & pairwise comparison to prioritize key attributes for assessing trainee selection to the program. Going forward, these attributes will be incorporated into the file review and interview portions of our admissions process. In addition to emphasizing these priority attributes in admissions, there are implications for other aspects of the program including curriculum and faculty development, as well as guiding the overall mission and vision for the Division. A similar process could be undertaken by other training programs seeking to identify priority attributes for admission to their programs.

## Introduction

Canadian child and adolescent psychiatry (CAP) subspecialty training constitutes a 2-year program with entry during the final year of a 5-year general psychiatry training program, for a total of 6 years of training following graduation from medical school. In 2012, the CAP subspecialty program at the University of Toronto (UT) was among the first to become a Royal College of Physicians and Surgeons of Canada (RCPSC) accredited training program. A total of 16 CAP programs have since been established and accredited across the country. There is a non-centralized application process, although, programs collaborate to determine shared timelines for application deadlines, interviews and offers. Each program admits one to seven applicants per year. The UT CAP program has been competitive, attracting as many as 15 applicants for 3–5 positions in the program. All applicants are typically highly accomplished and motivated residents. However, their career trajectories are often quite varied and residents enter the program with an array of strengths and interests. In the early days of the program, residents were selected based on a review of their application file (consisting of their CV, reference letters, and a personal statement) and two in-person semi-structured interviews. Although, there were 10 selection criteria, none of these was explicitly prioritized over the others, and thus they were subject to individual interpretation and implicit bias by file reviewers and interviewers. Moreover, these 10 criteria were identified by the selection committee without broader input from other stakeholders.

With increasing recognition of the importance of transparency and objectivity in the selection process (1), as well as a wish to attract diverse applicants from across Canada, in 2016 the Head of the Division of Child & Youth Mental Health (DCYMH) tasked the resident selection committee with modernizing their selection process by applying emerging best practices in selection, including: independent sampling, competency definition, multiple interviews, and relative ranking of candidates (2–4). The first step was to undertake a process of identifying the key applicant attributes to be considered in CAP trainee selection. The goals of this attribute identification quality-improvement (QI) exercise were 4-fold; (1) identification of an explicit set of attributes for use in the new selection protocol, (2) explicit descriptions of desired UT CAP applicant profiles, (3) mitigating sources of potential bias in the admissions process, and (4) distinguishing our program from others across the country, thus, distinctly positioning the UT CAP training program within the competitive national CAP training landscape (which we refer to as *branding*). Shappell and colleagues (5) define brand identity for postgraduate training programs as a “set of associations that defines a program, differentiates it from others in the specialty, and makes it relevant to specific target groups.” Although branding is often seen as a marketing goal, we also view it as a quality goal in order to develop overarching educational consistency across the many UT CAP teaching sites. Renewal of the CAP subspecialty selection process was seen to be an integral component of defining and establishing the UT CAP brand identity, thus, differentiating the UT CAP training program from others across the country (6) and fostering applicant's self-selection of the program. By defining key attributes and then incorporating them into the admissions process, we also sought to mitigate some sources of bias in the admissions process (7). Further, the identified priority attributes for resident selection would guide future CAP residency curriculum renewal to bring alignment across UT CAP selection and curriculum practices and ultimately the vision and mission for the DCYMH.

## Methods

### Phase 1: Consensus Building

Overall, an iterative design process was undertaken for this project. An initial list of potential attributes (e.g., leadership, scholarship, clinical skills, advocacy) was compiled during a brainstorming exercise led by project team members with the Executive of the DCYMH, at a half-day meeting dedicated to this purpose in March 2016. In June 2016, the project and goals were introduced to Divisional faculty and learners who attended the Divisional retreat. This event occurs annually and is attended by faculty and learners from the Division as an opportunity to reflect on and celebrate the previous academic year and plan for the year ahead. Educational theories, emerging concepts in medical education and evidence related to established best practices in selection processes were summarized for participants (2–4, 7–9). Following these didactic sessions, participants gathered in small groups to discuss the initial list of priority attributes and brainstorm further ideas. Over the summer & fall of 2016, further feedback was solicited from the DCYMH Residency Program Committee (RPC) and from key informants and stakeholders from within the DCYMH and the Department of Psychiatry. Subsequently, the project team reviewed the feedback that was gathered and identified eleven “branding attributes,” ten “generic attributes,” and seven “end products.” The team determined that the “end products” category could be eliminated for the purposes of this project, as these would be outputs of the training process rather than attributes to be assessed for selection into the program. Examples of “end products” included: academic psychiatrist, community-based psychiatrist, and researcher. “Branding attributes” were viewed as attributes that would contribute to brand identity (5), while “generic attributes” were defined as attributes that would be important for successful applicants to possess, but would not be specific to brand identity.

In order to further streamline attribute lists as well as maximize input and consensus regarding these lists, the project team undertook a consensus building exercise at the 2017 Divisional retreat. At that retreat, the selection committee's plan to move from two semi-structured interviews to four attributes-based Modified Personal Interviews (MPI) (3, 4) was summarized. It was reviewed that the priority attributes would serve as the criteria being assessed for resident selection, based on the file review and MPIs. A consensus-building activity was undertaken to determine the final list of attributes. For this activity, participants were divided into three groups and each group was provided with the lists of these words are an error eleven “branding attributes” and ten “generic attributes.” Each group was tasked with choosing their top five priority attributes from each list. To enhance engagement, the groups were given the freedom to determine how they achieved consensus. Each group then presented their lists and discussed why they chose to prioritize those specific attributes. As the groups presented their lists it became evident that there was already a great deal of overlap and consensus between the lists. Following each group's presentation and discussion of the notable commonalities and differences, participants were asked to anonymously vote on their preferred panels of attributes, resulting in the final panels of attributes chosen by vote (Table 1).

**Table 1:** Final lists of attributes chosen following consensus-building activity.

**Generic attributes**	**Branding attributes**
Integrity/ethics/morality	Scholarly/scholarship
Reflective practitioner	Advocate
Evidence informed/critical thinker	Leadership/capacity for leadership
Compassionate clinician	Clinical strength/expertise
Culturally competent	Capacity builder

### Phase 2: Ranking of Priority Attributes

The second phase of this project involved ranking in order of importance, via forced choice pairwise comparison (10), the attributes developed in Phase 1. For each of the attribute categories, respondents were randomly presented with each attribute pairing and were forced to choose one attribute from each pairing. The final rank order list of attributes was developed based on the number of times each attribute was chosen through all of the pairings. This pairwise comparison was completed electronically by members of the DCYMH following the 2017 retreat. As a means of increasing participation, project team members (CAK & MDH) visited eight of eleven Divisional teaching sites (hospitals and community mental health centres) to discuss the project at local medical staff meetings. These site visits provided further opportunity to discuss the overall goals of the project and to answer questions. Participants in the meeting were shown how to access the pairwise comparison survey and were encouraged to complete it following the meeting. A similar meeting was held with trainees enrolled in the UT CAP subspecialty training program. The pairwise comparison survey was also distributed electronically to members of the DCYMH so that it could be completed by anyone who was not in attendance at the informational sessions.

## Results

### Phase 1

With respect to the “generic attributes” lists, there was consensus among the groups at the Divisional retreat regarding the following attributes: Integrity/Ethics/Morality, Reflective practitioner, Evidence informed/Critical thinker, and Compassionate clinician. As such, a decision was needed only for the fifth attribute, with the options being Interprofessional, Collaborator, or Culturally Component. For the “branding attributes” lists there were three common attributes across all groups: Scholarly/scholarship, Advocate, and Leadership/Capacity for leadership. There was also agreement between two out of three groups for each of the remaining attributes. Consequently, there were only three attributes remaining from which the final two needed to be selected: Clinical strength/expertise, Capacity builder, and Innovator. Thus, the voting for the final panels of attributes only required choosing one final “generic” and two final “branding” attributes. The final lists of attributes determined through this process are presented in Table 1.

### Phase 2

The two lists of five attributes identified in Phase 1 were then ranked in order of importance using a paired-comparison forced choice survey. Half of the Divisional faculty and 40% of residents participated in the survey (Table 2). Of the faculty who responded, 61.6% identified as being based at an academic hospital site, while 34.2% identified as being based at a community-based site (16.4% community hospital, 17.8% children's mental health agency).

**Table 2:** Survey response rates.

**Respondent category**	**Number of respondents**	**Response rate**
DCYMH Faculty	73/145	50.3%
Current CAP trainees	4/10	40.0%
Total	77/155	49.7%

We report the results of the forced choice pairwise comparison for both the generic attributes (Table 3) and the branding attributes (Table 4). Of the generic attributes, integrity/ethics/morality (Attribute A in Table 3) was chosen the most frequently in each pairing. Similarly, clinical strength/expertise (Attribute I in Table 4) was the most frequently chosen in each pairing of the branding attributes. The forced choice pairwise comparison results were used to develop the final rank ordering for each list of attributes, which are presented in Table 5.

**Table 3:** Pairwise comparison results for “generic attributes.”

		**A**	**B**	**C**	**D**	**E**
Integrity/ethics/morality	A	-	A 68.83%	A 68.83%	A 53.25%	A 88.31%
Reflective practitioner	B	-	-	C 55.84%	D 61.04%	B 83.12%
Evidence informed/critical thinker	C	-	-	-	C 51.95%	C 81.82%
Compassionate clinician	D	-	-	-	-	D 85.71%
Culturally competent	E	-	-	-	-	-

*Respondents were forced to choose between each pairing. The number seen is the percentage of respondents who chose the attribute listed from the pairing. (e.g., in the pairing integrity/ethics/morality vs. reflective practitioner or A vs. B, 68.83% of respondents chose integrity/ethics/morality or A.)*.

**Table 4:** Pairwise comparison results for “branding attributes.”

		**F**	**G**	**H**	**I**	**J**
Scholarly/scholarship	F	-	F 50.65%	H 54.55%	I 83.12%	J 50.65%
Advocate	G	-	-	H 55.84%	I 85.71%	J 57.14%
Leadership/capacity for leadership	H	-	-	-	I 80.52%	H 51.95%
Clinical strength/expertise	I	-	-	-	-	I 78.95%
Capacity builder	J	-	-	-	-	-

*Respondents were forced to choose between each pairing. The number seen is the percentage of respondents who chose the attribute listed from the pairing. (e.g., in the pairing scholarly/scholarship vs. advocate or F vs. G, 50.65% of respondents chose scholarly/scholarship or F.)*.

**Table 5:** Results of pairwise comparison – relative rankings.

**Relative ranking**	**Generic attributes**	**Branding attributes**
1.	Integrity/ethics/morality	Clinical strength/expertise
2.	Evidence informed/critical thinker	Leadership/capacity for leadership
3.	Compassionate clinician	Capacity builder
4.	Reflective practitioner	Scholarly/scholarship
5.	Culturally competent	Advocate

## Discussion/Conclusions

We used an interactive consensus building approach to develop a list of key applicant attributes, and then a pair-wise comparison approach to rank these attributes for use in resident selection. This project has shown that such an approach is feasible, thus allowing for collaborative co-construction of priority attributes. These priority attributes can now be used to transparently describe desired applicant profiles, thus achieving the primary goals of this project. Information about attributes being sought can be listed in recruitment materials, including the program webpage, and incorporated into the admissions process. Some attributes may be best suited for assessment via file review, some through interview, and some through both processes. Assessment of attributes will need to be operationalized so they can be assessed when reviewing files and/or through interviews (e.g., each interview focusing on a subset of the attributes). Although, the attributes may be perceived as subjective, the interview allows for focus on applicant experiences that reflect these attributes in the delivery of mental health care for children, youth, and their families. By developing transparent criteria that can be used across the selection process, we can mitigate potential sources of bias (11, 12), an additional goal of this project. Previously different file reviewers/interviewers may have implicitly prioritized different attributes, leading to variability between assessors and across years. With transparent lists of priority attributes constituting preferred applicant profiles, admissions assessments are based explicitly on commonly valued and defined criteria (2, 8), rather than the unperceived values and biases of individual assessors.

The final goal of this project was to distinctly position as a brand the UT CAP training within the competitive national CAP training landscape. Rather than a top-down approach dictated by a few individuals, the involvement of members of the entire Division in developing these attributes was a critical aspect in gathering input that accounts for the organizational culture and wishes of its members (6). There is no national centralized system for admissions across the 16 Canadian CAP programs. By explicitly defining the attributes being sought in incoming UT CAP residents, we are paving the way toward the development of an explicit brand that differentiates the UT CAP program from other CAP programs across the county. We acknowledge that other programs may seek overlapping qualities in their applicants; however, we are the first CAP program in Canada to explicitly identify and recruit for specific attributes. In doing so, the program stands out as one that is interested in recruiting from the national candidate pool and not solely from the local pool of potential candidates. From the applicant perspective, future applicants can be clear about the priorities of the program, thus allowing them to make informed decisions about applying to and accepting a position in the program. Given the high number of applicants for limited positions, this opportunity to ensure fit is particularly important.

Interestingly, the attribute of “clinical strength/expertise” was labeled by participants as a branding attribute. Later discussions by the project team led to the realization that perhaps this attribute should have been considered a generic attribute, as all training programs would intend to train excellent clinicians and so this attribute does not necessarily contribute to brand identity *per se*. Given UT's history as an institution with a significant research focus, it was somewhat surprising that scholarship was ranked fourth out of the five branding attributes, as it might have been anticipated to be more highly ranked. We note that we strove to involve respondents from a variety of Divisional sites, including affiliated community-based sites, which may explain the relative value placed on clinical abilities over scholarship in this iteration of the ranking. This serves as a lesson for those considering a brand development process that is collaborative and influenced by organizational culture – that it is important to gather a wide range of opinions and use established methodology such as a paired comparison to develop the actionable brand. Finally, the idea of leadership ranking so highly was initially questioned by some faculty members, who were concerned that selecting for future leaders would result in narrowing applicants to those with administrative or academic leadership aspirations. However, we would argue that the operationalization of leadership can and should be sufficiently broad as to enhance diversity and richness by selecting applicants who have the potential for formal and informal leadership in a wide variety of spheres including, but not limited to, research, advocacy, systems change, education, and equity. We anticipate that many graduates may not end up in formal leadership roles, but will still benefit from leadership skills that they can apply within their practices and the health care system.

The lists of attributes we identified will have broader impacts beyond trainee selection. In addition to selecting trainees who are already strong in these priority attributes, the program will emphasize these attributes in the curriculum, thus moving toward brand implementation (5). As such, going forward there will be a need for both curricular renewal and faculty development in relation to the identified attributes. Moreover, the attributes reflect the co-constructed priorities of the current faculty and learners in the Division. The attributes have since been incorporated into the Division's mission & vision statement that are articulated on the Divisional website, thus highlighting both internally and externally their value to the leadership and contributing to brand image (5).

Although, this project allowed for a great deal of collaboration and consensus building within the DCYMH, including physician and non-physician members, one potential limitation is that it did not involve individuals from outside the Division. It would have been valuable to include voices from within the broader Department of Psychiatry or the Faculty of Medicine. Incorporating viewpoints of persons with lived experience in defining the attributes that they would expect in future child and adolescent psychiatrists is also important. In future iterations of this project, it would add value to expand the collaborative process to include key stakeholders, such as family advocates and persons with lived experience, to further enhance the applicability of this work.

The collaborative consensus building approach that we undertook to develop our priority attributes can be applied and modified as needed at other institutions. As the project was conducted with minimal budget and resources, many other institutions should be able to replicate it. As the program's brand continues to develop, this work should be considered the first part of an iterative process that is repeated over time as priorities evolve. Future directions include operationalizing the attributes as they are applied to the admissions process, as well as measuring the reliability of assessing them. Input from residents and eventually from graduates will be valuable in understanding the influence of the attributes on how applicants view our program and the experience of residents trained in it.

## Data Availability Statement

The raw data supporting the conclusions of this article will be made available by the authors, without undue reservation.

## Ethics Statement

Ethical review and approval was not required for the study on human participants in accordance with the local legislation and institutional requirements. Written informed consent for participation was not required for this study in accordance with the national legislation and the institutional requirements. The REB at the University of Toronto deemed this project a Quality Improvement (QI) project not requiring formal REB review or approval. It is thus noted that any conclusions made based on this project were not gained through research but through a quality improvement project carried out in a local context.

## Author Contributions

CAK led project and writing or main manuscript, editing, and submission. MDH oversaw and advised on project and manuscript, provided guidance, and editing. All other authors contributed equally to the idea generation, suggestions, and editing of the manuscript.

## Conflict of Interest

The authors declare that the research was conducted in the absence of any commercial or financial relationships that could be construed as a potential conflict of interest.
